# A Robust Computational Technique for Model Order Reduction of Two-Time-Scale Discrete Systems via Genetic Algorithms

**DOI:** 10.1155/2015/615079

**Published:** 2015-03-08

**Authors:** Othman M. K. Alsmadi, Zaer S. Abo-Hammour

**Affiliations:** ^1^Department of Electrical Engineering, The University of Jordan, Amman 11942, Jordan; ^2^Department of Mechatronics Engineering, The University of Jordan, Amman 11942, Jordan

## Abstract

A robust computational technique for model order reduction (MOR) of multi-time-scale discrete systems (single input single output (SISO) and multi-input multioutput (MIMO)) is presented in this paper. This work is motivated by the singular perturbation of multi-time-scale systems where some specific dynamics may not have significant influence on the overall system behavior. The new approach is proposed using genetic algorithms (GA) with the advantage of obtaining a reduced order model, maintaining the exact dominant dynamics in the reduced order, and minimizing the steady state error. The reduction process is performed by obtaining an upper triangular transformed matrix of the system state matrix defined in state space representation along with the elements of *B*, *C*, and *D* matrices. The GA computational procedure is based on maximizing the fitness function corresponding to the response deviation between the full and reduced order models. The proposed computational intelligence MOR method is compared to recently published work on MOR techniques where simulation results show the potential and advantages of the new approach.

## 1. Introduction

Model order reduction (MOR) of multi-time-scale systems has been an important subject area in control engineering for many years [1, 2]. In many industrial control systems, simple controllers are preferable. However, derivation of the mathematical model often leads to detailed description of a complex model in the form of high order differential equations [2]. Due to this point of view along with other different design objectives, model order reduction has been an active research area in the control society since the 1960s where a large number of model order reduction methods have been introduced in literature for single input single output (SISO) as well as MIMO type systems. The reduction operation is to search for the possibility of finding some lower order equations of the same type that may be considered to adequately reflect the dominant characteristics of the original system. The objective of simplification is to obtain a low order model of the existing high order model such that both are equivalent in terms of system response and being close to each other in some physical representation means. Model reduction problems have attracted much attention in recent years; for example, the model reduction problem has been investigated using artificial neural networks [3], genetic algorithms [4], and invasive weed optimization [5]. It was also used in nonlinear systems [6], gain scheduling [7], linear time-varying systems [8, 9], and linear parameter-varying systems [10].

To obtain a model of lower order, a significant number of methods have been proposed in recent and earlier years, some for continuous time systems [3–5] and some for discrete-time systems [1, 11–15]. Some methods, such as model order reduction by matching Markov parameters [16], were introduced to ensure stability of the reduced order model. A popular technique for obtaining reduced order models is the Krylov subspace [17]; however, stability of the reduced model is not guaranteed. Another important group of reduction algorithm is the eigenvalue preservation technique [3–5, 11] where important eigenvalues of the system are retained to find suitable lower order models.

A numerous number of MOR methods are available for continuous systems, but very few have been devoted to the discrete-time systems MOR. The discrete-time system MOR may be performed in two different ways. The first one is performed based on transforming a continuous time model into another form using different types of transformation as seen in [6, 18]. In this group of MOR, the reduction process is completely performed in the continuous time form. The discrete reduced order model is then obtained by the corresponding inverse transformation of the continuous time reduced model. The second method for obtaining a discrete reduced order model, which is known as a direct method [14], is deriving the discrete reduced order model directly without using any type of transformation. Some of these methods perform the MOR using canonical expansion of *z*-transfer function and stable optimal methods [13, 19], power decomposition and system identification [20], and multipoint step response matching [21]. New optimization techniques, particle swarm optimization [22], and artificial neural networks [11] have also been introduced for MOR of discrete-time systems.

GA-based MOR, on the other hand, has received some of the researchers' attention as well. Recently, Ponda et al. [23] employed a particular swarm optimization technique to obtain a reduced order model of SISO large scale linear systems. Their technique is based on the integral square error (ISE). Vishwakarma and Prasad [24] proposed a mixed method for reducing the order of large-scale linear systems. They have synthesized the denominator of the reduced order transfer function using modified pole clustering while the coefficients of the numerator elements are computed using GA. Parmar et al. [25] presented a technique for model order reduction using GA for SISO linear time systems. They have focused on obtaining a reduced order model that maintains stability and retains the steady state value. In spite of the methods available in literature, each method has advantages and disadvantages when tried on a particular system. In addition to that, no approach always gives the best results for all systems. It is important to mention that GAs have also been used for model system identification, where order and parameters are set to be determined, as we have investigated in [26, 27]. In this paper, however, and as motivated by the singular perturbation method which has the characterization of multi-time-scale systems, the GA procedure is performed with the advantages of retaining the exact dominant dynamics in the designed model, obtaining a new robust model with a lower order, and maintaining a minimum steady state response error.

The work in this paper is organized as follows: Section 2 presents problem formulation of the discrete full and reduced order models. In Section 3, the genetic algorithm approach for MOR of multi-time-scale discrete systems is presented. Illustrative examples utilizing the new approach along with simulation comparative results of different MOR techniques are presented in Section 4.  Section 5 presents an overall conclusion of the proposed MOR method.

## 2. Problem Formulation

In this paper, MOR is investigated for discrete LTI systems of both SISO and MIMO type models. For SISO systems, a transfer function model is used, while the state space representation is used for MIMO systems.

For the SISO systems, consider the discrete-time system described by

(1) yk+a1yk−1+a2yk−2+⋯+anyk−n =b0uk+b1uk−1+⋯+bn−uk−n−,

where *u*(*k*) is the input and *y*(*k*) is the output of the system at the *k*th sampling instant. Equation (1) can be written in the form of a pulse transfer function as

(2) $$\begin{array}{c} G\left(z\right)=\frac{Y\left(z\right)}{U\left(z\right)}=\frac{b_{0}z^{\overset{-}{n}}+b_{1}z^{\overset{-}{n}-1}+\cdots +b_{\overset{-}{n}}}{z^{n}+a_{1}z^{n-1}+\cdots +a_{n}} \end{array}$$

with $\overset{-}{n}\leq n$. The characteristic polynomial contains the system dominant and nondominant poles (distinct, repeated, or complex) where their number, *n*, is referred to as the model order. The corresponding desired reduced *r*th order model is given by

(3) $$\begin{array}{c} G_{r}\left(z\right)=\frac{Y\left(z\right)}{U\left(z\right)}=\frac{b_{0}z^{\overset{-}{r}}+b_{1}z^{\overset{-}{r}-1}+\cdots +b_{\overset{-}{r}}}{z^{r}+a_{1}z^{r-1}+\cdots +a_{r}}, \end{array}$$

where some of the coefficients *a*_*i*_  (*i* = 1,2,…, *r*) and $b_{i}\;\;(i=0,1,2,\ldots ,\overset{-}{r})$ may be zeros as long as $\overset{-}{r}\leq r$. For the MIMO systems, consider the following *n*th order discrete-time system:

(4) $$\begin{array}{c} x\left(k+1\right)=Ax\left(k\right)+Bu\left(k\right), \end{array}$$

(5) $$\begin{array}{c} y\left(k\right)=Cx\left(k\right)+Du\left(k\right), \end{array}$$

where *k* is the time index, *x* ∈ ℜ^*n*^ is the state vector, *u* ∈ ℜ^*p*^ and *y* ∈ ℜ^*m*^ are the input and output vectors, respectively, and *A* ∈ ℜ^*n*×*n*^, *B* ∈ ℜ^*n*×*p*^, *C* ∈ ℜ^*m*×*n*^, *D* ∈ ℜ^*m*×*p*^ are matrices of appropriate dimensions with *n*, *p*, and *m* being the system order, number of inputs, and number of outputs, respectively. The corresponding desired reduced *r*th order model is obtained as follows:

(6) $$\begin{array}{c} x_{r}\left(k+1\right)=A_{r}x_{r}\left(k\right)+B_{r}u\left(k\right), \end{array}$$

(7) $$\begin{array}{c} y_{r}\left(k\right)=C_{r}x_{r}\left(k\right)+D_{r}u\left(k\right), \end{array}$$

where *x*_*r*_(*k*) is *r*-state vector, *y*_*r*_(*k*) is the reduced order model output, and *A*_*r*_, *B*_*r*_, *C*_*r*_, and *D*_*r*_ are matrices with appropriate dimensions.

## 3. Genetic Algorithms with MOR

GAs are based on principles inspired from the genetic and evolution mechanisms observed in natural systems. Their basic principle is the maintenance of a population of solutions to the problem that evolves in time. They are based on the triangle of genetic reproduction, evaluation, and selection [12]. Genetic reproduction is performed by means of two basic genetic operators: crossover and mutation. Evaluation is performed by means of the fitness function that depends on the specific problem. Selection is the mechanism that selects parent individuals with probability proportional to their relative fitness.

In this paper, using the computational intelligence of GA, we will obtain the reduced order model based on only the dominant dynamics of the system. For dynamic decoupling, the GA will set the reduced order model state matrix *A*_*r*_ in the modal form where all of the selected eigenvalues (system dynamics), real and/or complex, are placed on the diagonal. Thus, the reduced order model state matrix *A*_*r*_, in (6), is designed to have the following decoupling format:

(8) $$\begin{array}{c} A_{r}=\left[\begin{array}{ccccccccc} \lambda_{1} & a_{12} & a_{13} & a_{14} & & \cdots & & & a_{1r}\\ 0 & \lambda_{2} & a_{23} & a_{24} & & \cdots & & & \\ & \ddots & \ddots & \vdots & & & & & \\ \cdot & & 0 & \lambda_{\overset{-}{b}} & & & & & \vdots \\ \cdot & & & 0 & \sigma_{1} & \alpha_{1} & & & \\ \cdot & & 0 & & -\alpha_{1} & \sigma_{1} & & & \\ & & 0 & & & 0 & \ddots & & a_{(r-2)r}\\ & & & & & & 0 & \sigma_{\overset{-}{p}} & \alpha_{\overset{-}{p}}\\ 0 & & \cdot & \cdot & \cdot & & 0 & -\alpha_{\overset{-}{p}} & \sigma_{\overset{-}{p}} \end{array}\right], \end{array}$$

where the original system dominant poles (real and/or complex) are preserved in the diagonal, seen as *λ*_*i*_, $i=1,2,\ldots ,\overset{-}{b}$ (real) and *σ*_*i*_ ± *α*_*i*_, $i=1,2,\ldots ,\overset{-}{p}$ (complex). Notice that, for this reduced order model, $r=(\overset{-}{b}+2\overset{-}{p})<n$. To insure that the dominant poles are preserved in the reduced order model and for further order reduction, the following condition is satisfied:

(9) $$\begin{array}{c} \lambda_{\text{dominant}}\left.:=\right|\left|\lambda_{1}\right|>\left|\lambda_{2}\right|>\cdots >\left|\lambda_{\overset{-}{b}}\right|,\;\left|\lambda \right|>\left|\sigma \right|,\\ \left|\sigma_{1}\pm \alpha_{1}\right|>\left|\sigma_{2}\pm \alpha_{2}\right|>\cdots >\left|\sigma_{\overset{-}{p}}\pm \alpha_{p}\right|. \end{array}$$

Take into account that if |*λ*_*i*_| < |*σ*_*i*_ ± *α*_*i*_|, then (9) is to be redefined accordingly if necessary. For simplicity, the modal form is chosen, which implies that all elements seen in (8) as *a*_*ij*_ (*i* and *j* = 1,…, *r*) are set to zero.

The GA will determine the parameters of the *B*_*r*_ and *C*_*r*_ (and *D*_*r*_ if necessary) in (6) and (7). Hence, the total number of elements that the GA will need to find is given by

(10) $$\begin{array}{c} n_{p}=n_{r}\cdot m+p\cdot n_{r}. \end{array}$$

It is to be noted that all of the parameters that the GA will have to find are restricted to be real values. Now, based on the number of unknown parameters (*n*_*p*_), the GA creates a population of individuals, where each parameter is basically an individual in this population. The population consists of different “sets” of solutions. Each solution set is called a chromosome, which contains *n*_*p*_ individuals. Given a population size (*n*_pop_), a matrix consisting of *n*_pop_ rows is formed with each row containing one set of solutions for the unknown parameter values. This would result in a matrix containing *n*_pop_ × *n*_*p*_ elements. Each element in this matrix contains a value pertaining to one unknown parameter, where each row presents one set of solutions.

The genetic algorithm used in this work will operate as follows.

### 3.1. Initialization

An initial population comprising *N*_*p*_ individuals is randomly generated. The GA type used in this paper is a binary genetic algorithm, where each value in the solution set consists of a number of bits (genes). The number of bits used to encode each numerical value depends on three variables, lower parameter bound (*p*_*l*_), upper parameter bound (*p*_*u*_), and accuracy (*ϕ*). Hence, the number of bits used is defined as follows [30]:

(11) $$\begin{array}{c} n_{\text{bits}}=\frac{\text{lo}{\text{g}}_{10}\left(\left(p_{u}-p_{l}\right)/\phi \right)}{\text{lo}{\text{g}}_{10}\left(2\right)}. \end{array}$$

Given that each parameter value will consist of *n*_bits_ number of bits, each solution set will consist of *n*_*b*,*t*_ = *n*_*p*_ · *n*_bits_ bits. This represents one row of the entire *n*_pop_ × *n*_*b*,*t*_ matrix.

**Figure 1:**
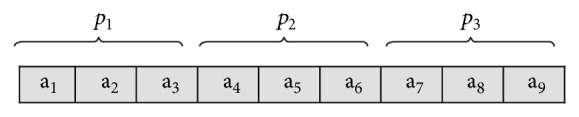
A set of individuals in a GA chromosome.

The GA starts by randomly initializing a binary matrix with *n*_pop_ rows and *n*_*b*,*t*_ columns. Each row (set of solutions) is made up of multiple values decoded into binary and placed next to each other as illustrated in Figure 1 for one chromosome. This chromosome consists of three parameters (individuals) with each individual being made of three genes. These genes (bits) can be later decoded back into decimal values, which in return provide the desired parameters' values.

The initial population matrix consists of random numbers within the lower and the upper bounds of the parameters. The population will be split into rows (chromosomes), each constituting one solution set. These solution sets are each taken to have their fitness evaluated, as seen next.

### 3.2. Evaluation

The fitness, a nonnegative measure of quality, is used as a measure to reflect the degree of goodness of the individual and is calculated for each individual in the population. This measure of quality is calculated based on minimizing the following related cost function:

(12) $$\begin{array}{c} e\left(k\right)=y\left(k\right)-y_{r}\left(k\right) \end{array}$$

which is the deviation between the full and reduced order models' responses given in (5) and (7). The fitness for each chromosome (solution set) is then evaluated as follows [31]:

(13) $$\begin{array}{c} \text{Fitness}=\frac{1}{1+\text{MSE}}\times 100\%, \end{array}$$

where

(14) $$\begin{array}{c} \text{MSE}=\frac{1}{N}\overset{N}{\underset{i=1}{\sum }}{\left[y\left(i\right)-y_{r}\left(i\right)\right]}^{2}, \end{array}$$

where MSE is the mean-square-error and *N* is the number of elements in the output vector(s). The higher this fitness is, the closer the reduced model is to mimic the original model.

### 3.3. Selection

In the selection process, chromosomes are chosen from the current population to enter a mating pool devoted to the creation of new children (offspring) for the next generation. The chance of a given individual to be selected to mate is proportional to its relative fitness That is, the larger the fitness value of a chromosome, the higher the probability of the chromosome to contribute one or more children in the next generation. First, the population fitness and associated chromosomes are ranked from highest fitness to lowest fitness. Then, only the best are selected to continue, while the rest are left. The selection rate (*X*_*r*_) is a rank based ratio, which is the percentage of the best rank individual that should move to the mating pool where pairs of the mating pool are selected for crossover process. Deciding how many chromosomes to keep is somewhat arbitrary. Letting only a few chromosomes survive to the next generation limits the available genes in the offspring (children). Keeping too many chromosomes gives bad performers a chance to contribute their traits to the next generation. In the proposed algorithm, an initial selection rate *X*_*r*_ = 0.60 was used and the top 60% fitness chromosomes were placed in a mating pool.

There are several methods for choosing the chromosome pairs to be mated from the mating pool. In the proposed algorithm, random pairing was chosen, which uses a uniform random number generator to select chromosomes that enter the mating pool from kept chromosomes. The algorithm randomly chooses chromosomes from the mating pool for mating, while making sure that all pairs are unique. After the chromosome pairs are chosen, the next operation will be crossover.

### 3.4. Crossover

**Figure 2:**
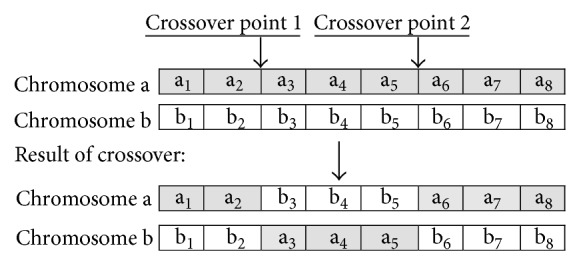
Chromosomes random crossover operation.

Crossover provides the means by which valuable information is shared among the population. In the crossover operation, a pair of chromosomes is mated to produce two offspring in the process that inherit genes from their parents. To perform crossover, the chromosomes need to be in their gene format (i.e., binary representation). The two parent chromosomes are crossed over at random points to segment each chromosome into a number of parts. These parts of each chromosome pair are swapped between each other to produce two new chromosomes, the offspring. This is shown in Figure 2.

Crossover is not applied to all pairs of chromosomes selected for mating. For every parent chromosome pair, a crossover rate decides whether or not crossover occurs. If crossover is not applied, children are produced simply by duplicating the parents. This gives each chromosome a chance of passing on its genes without the disruption of crossover. In the proposed algorithm, the crossover rate is set to 0.80, meaning that 80% of parent pairs produce offspring. The number of crossover points for the parent chromosomes is chosen as a random integer for each generation. As a crossover result, a new children population will be performed with the same size as the parent population.

### 3.5. Mutation

Mutation is often introduced to guard against premature convergence. Generally, over a period of several generations, the gene pool tends to become more and more homogeneous. Therefore, further mutation is introduced to the offspring to guarantee that the offspring will have new qualities while retaining a similar overall structure. Mutation is applied to each child individually after crossover, where it randomly flips any bit (gene) with a small mutation probability (between 0.1 and 0.001). Mutation provides a small amount of random search to guard against premature convergence. In the proposed methodology, the mutation rate is set to 0.0125.

### 3.6. Replacement

Replacement operation takes place once the crossover of all parent chromosome pairs is performed. The parent population is totally or partially replaced by the children population depending on the replacement scheme used. This completes the life cycle of the population. At this stage, the population is ready to enter the next generation and undergo a new round genetic operation. The decision in which offspring replaces which population individual is made based on the fitness evaluation. Fitness is evaluated for all the resulting offspring as well as for all of the original population individuals and a replacement factor decides how many of the offspring with the highest fitness values are to replace the main population individuals. In the proposed approach, a replacement factor of 0.70 is used. This process continues until reaching the end of the population size or best desired fitness.

### 3.7. Termination

The GA is terminated when some convergence criterion is met. The termination condition could be considered as specified fitness value, reaching maximum number of generations, or a set progress limit. As one generation has gone by, depending on the genetic algorithm's termination condition, the algorithm could stop any time and identifies the chromosome with the highest fitness value as the optimal solution set. On the other hand, it may repeat the entire process from the selection procedure to continue another generation of the genetic algorithm. The termination condition for the proposed algorithm is reaching a fitness value of 99.999% and an average fitness value of at least 99.9% in the entire population set. When the algorithm terminates, the highest fitness chromosome is distributed across the *B*_*r*_, *C*_*r*_, and *D*_*r*_ matrices to produce the reduced order model.

## 4. Illustrative Examples

In this section, we consider a few discrete system examples, which have been investigated by different researchers in recent years. The examples are given as SISO and MIMO linear time invariant systems. Results of investigations are compared with the existing ones to show the potential of the new approach.

**Example 1 .**

As a first example, we consider the following 7th order SISO system investigated by Telescu et al. [32] given as

**Figure 3:**
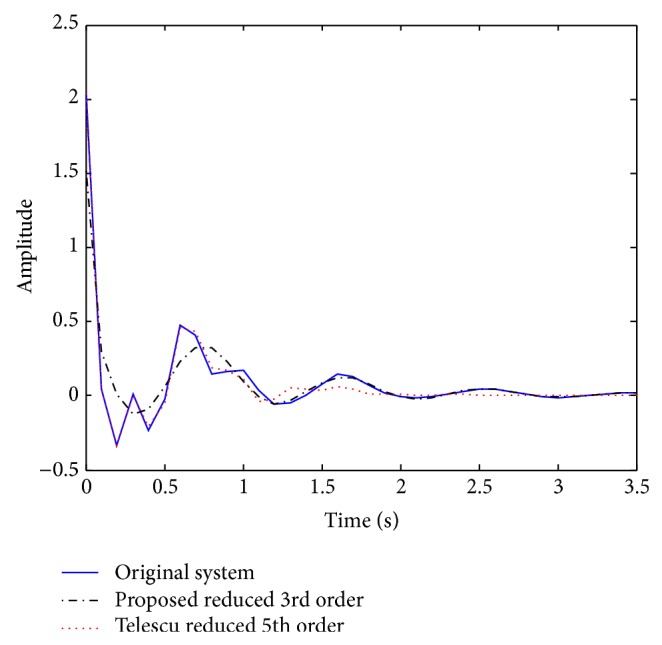
Impulse responses for the full and reduced order models.

(15) Gz=2.0434z7−4.98255z6+6.57z5−5.8189z4  + 3.636z3−0.00088z2−1.4105z+0.2997 ·z7−2.466+3.433z5−3.333z4  + 2.546z3−1.584z2+0.7478z−0.252−1

with system dynamics (poles): [0.8913, 0.6843 ± 0.5820*i*, 0.2988 ± 0.7574*i*, −0.1987 ± 0.6993*i*]. Telescu et al. [32] used the Laguerre functions for their proposed method and obtained a 5th order reduced model:

(16) Gr(z)=2.043z5−3.057z4+2.195z3−1.545z2+0.8617zz5−1.518z4+1.270z3−1.032z2+0.7539z−0.3156

with poles given as [0.8320, −0.2318 ± 0.7612*i*, 0.5748 ± 0.5183*i*], which are unrelated to the original system dynamics. On the other hand, our first advantage of the proposed technique is that a lower dimension (3rd order) than the Telescu reduced model was obtained:

(17) $$\begin{array}{c} G_{r}\left(z\right)=\frac{1.51511z^{3}-3.12377z^{2}+2.40413z-0.63117}{z^{3}-2.2598z^{2}+2.0266z-0.71915} \end{array}$$

with poles given as [0.8913, 0.6843 ± 0.5820*i*], which are exactly the dominant dynamics of the full order system as seen above, and this is our second advantage. The result was obtained for a population of 1000 and 300 generations with a fitness of 99.992. To investigate the system behavior, the full and reduced order models were excited by an impulse input (as performed by Telescu) with results of simulation shown in Figure 3, which shows our third advantage as the propose 3rd order reduced model response converges in about one second, which is much faster than the Telescu's response.

**Figure 4:**
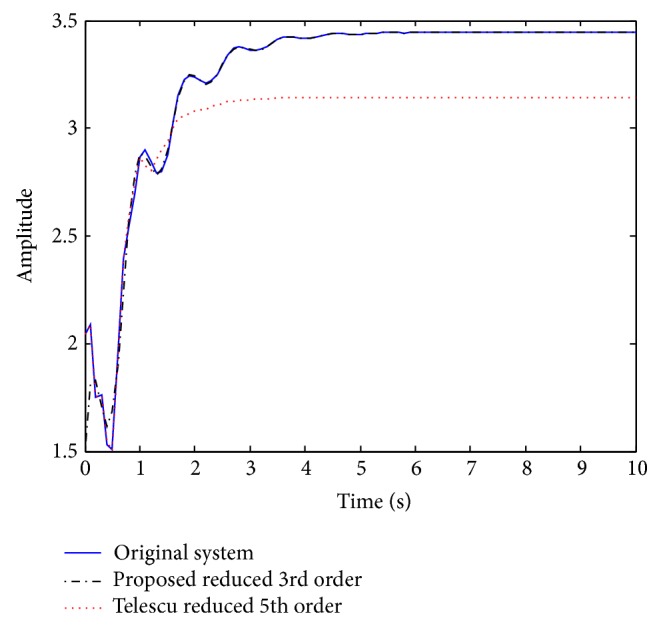
Step responses for the full and reduced order models.

Our fourth advantage is clearly seen when simulating the full and reduced models to a step input. As seen in Figure 4, Telescu et al. [32] 5th order model has a huge steady state error while the proposed 3rd order model's error can barely be seen at the steady state which shows the robustness of the proposed method.

**Example 2 .**

In this example, we consider the following 8th order SISO system investigated by Yadav et al. [29]:

(18) Gz=0.1625z7+0.125z6−0.0025z5+0.00525z4  − 0.02263z3−0.00088z2+0.003z−0.000413 ·z8−0.6307z7−0.4185z6+0.078z5−0.057z4  + 0.1935z3+0.09825z2−0.0165z+0.00225−1

with system dynamics: [0.8797 ± 0.2442*i*, −0.0542 ± 0.6558*i*, −0.5875 ± 0.0959*i*, 0.0773 ± 0.1078*i*]. Using the proposed method, the dominant dynamics [0.8797 ± 0.2442*i*] are preserved in the reduced order model which is given by

(19) $$\begin{array}{c} G_{r}\left(z\right)=\frac{0.021129z^{2}+0.12259z-0.063666}{z^{2}-1.7594z+0.83347}. \end{array}$$

For result evaluation, the proposed reduced order model was compared with recent research for MOR, that is, artificial bee colony [28], differential evolution optimization algorithm, and real coded genetic algorithm [33]. Hence, we first compare for dominant dynamics of the reduced order models with results obtained as in Table 1. As can be seen, the proposed method provides the dominant dynamics of the full order model retained exactly in the reduced order, while in the other methods, the reduced order dynamics are close to the full order model, but not as close as our proposed method's results.

**Figure 5:**
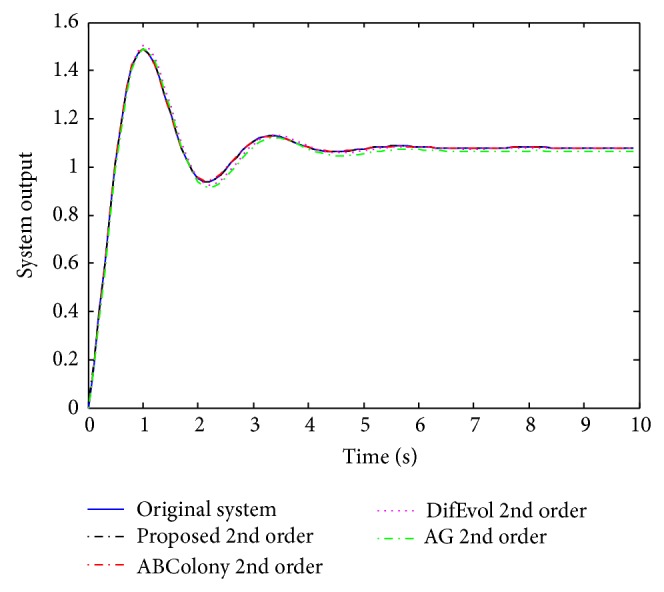
Step responses for the 8th and reduced 2nd order models.

**Figure 6:**
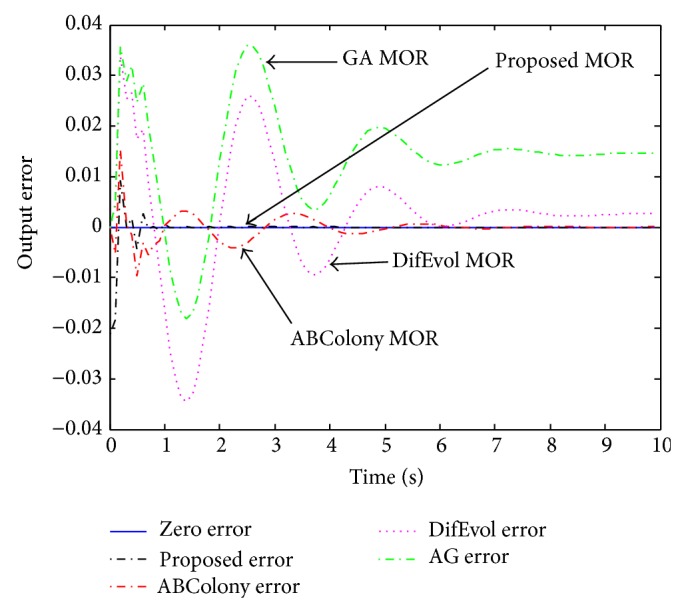
Error comparison of the reduced order models with the full order.

As a second comparison, the full and reduced order models were simulated for a step input with results as seen in Figure 5. Observing the results in Figure 5, we can see that the 2nd order reduced model of all methods seems to be close to the original full order model. However, when we take a closer look, we see the differences very clearly as present in Figure 6. In this figure, it is seen that the proposed approach provides the least error among all. The error is seen a little at the beginning and then, at about one second, the error becomes very close to zero.

**Example 3 .**

In this example, we consider the 5th order MIMO discrete system investigated by Li [2] as given by the following state space model:

(20) xk+1 =0.50340.1768−0.2340−0.14060.58140.00960.5498−0.0362−0.67442.24960.03370.25460.0984−0.40511.3599−0.27090.14700.32490.04840.6356−0.09090.04910.1075−0.10190.5681x(k)  +0.33060.17000.89510.34420.54870.21430.87480.88210.52170.4479uk,yk=3.0622−0.9986−0.71266.4339−10.42913.0396−0.9913−0.70735.2369−8.4887xk +0000uk.

**Figure 7:**
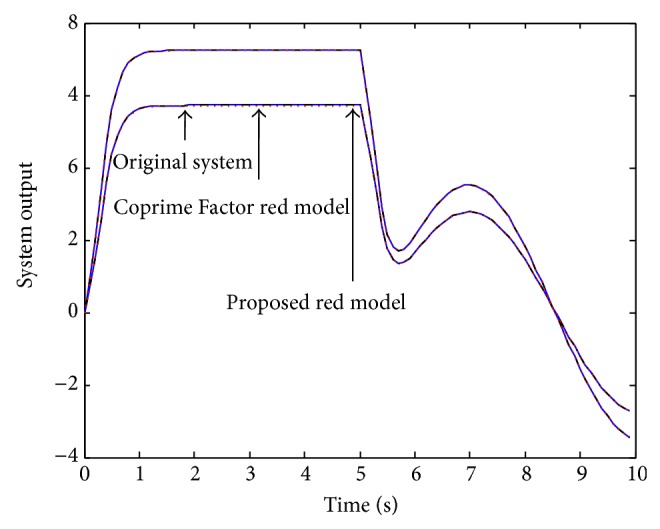
Full and reduced order model's responses for mixed input signals.

**Figure 8:**
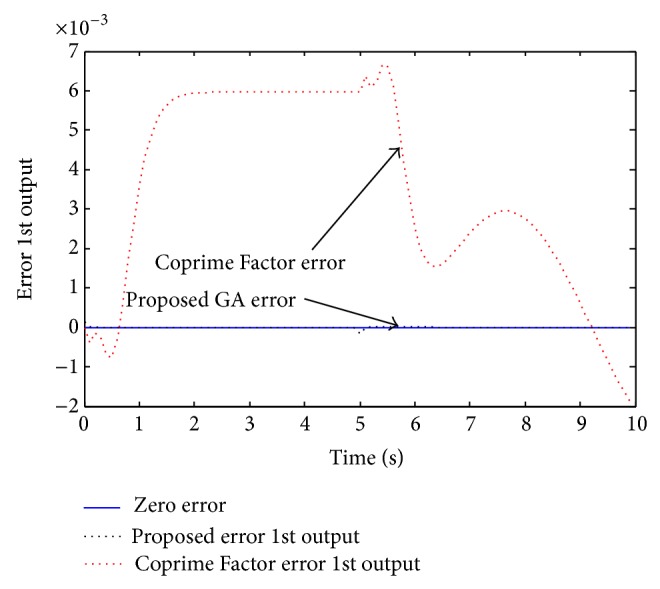
Error of the reduced order models for the system first output response.

**Figure 9:**
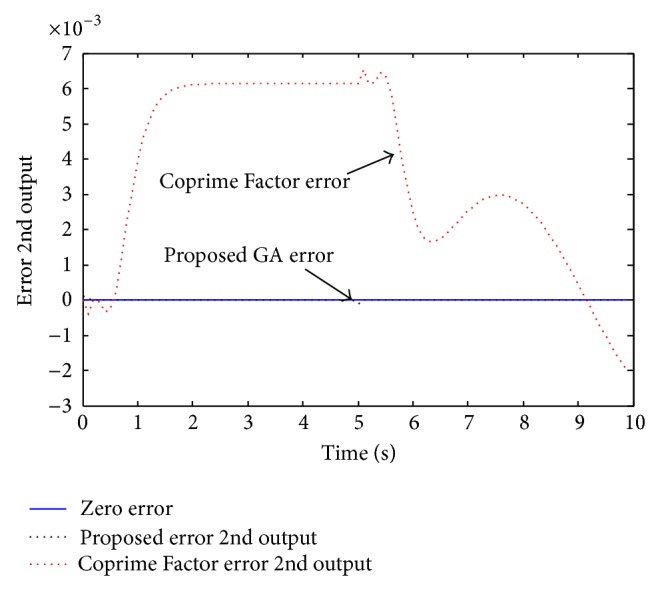
Error of the reduced order models for the system second output response.

This system is given with the dynamics [0.5614, 0.5008, 0.3389, 0.2295, 0.1375]. Li performed the Coprime Factor MOR and obtained the following 4th order reduced model:

(21) xrk+1=0.38300.0865−0.37710.7548−0.16330.6255−0.01570.1556−0.67310.51500.3777−0.2194−0.36920.28320.00940.2388xrk +1.05720.0349−0.2642−0.5105−0.6643−1.57900.98640.0412uk,yk=−0.2745−0.0669−0.6423−0.2425−0.2725−0.0664−0.5228−0.1973xrk +0000u(k)

with poles [0.5567, 0.5004, 0.3385, 0.2294], which are close to the full order model, but not the exact values. Using the proposed approach, the following 4th order reduced model was obtained:

(22) xrk+1=0.561400000.500800000.338900000.2295xr(k) +0.42850.26280.41570.25510.34050.23480.31110.2671uk,yk=29.7314−20.0311−37.938227.072326.5158−20.4912−28.500321.6793xrk +10−30.0061−0.15800.0046−0.1191u(k)

with poles [0.5614, 0.5008, 0.3389, 0.2295], which are the exact dominant dynamics of the original model. Hence, the superiority of the proposed method is clearly seen. Next, we investigate for quality performance of the new method compared with the Li approached reduced order model. Simulating the full and reduced order models for a mixed type signal, the two system output responses are presented in Figure 7. As seen in this figure, the two reduced order models seem to perform with same quality. Therefore, in order to observe the differences between the proposed and existed methods, we present the response accuracy error, as seen in Figures 8 and 9 for the two output responses.

In Figures 8 and 9, notice that the Coprime Factor method response errors are much far from zero when compared with the proposed method response errors. The response errors of the proposed GA approach can barely be seen as they are almost zeros, which clearly shows the superiority of the proposed approach.

As seen in the three previous examples, the advantages of the new approach are present in all three of them. That is, the reduction process provides new reduced order models, maintains the exact dominant dynamics of the original model in the reduced order model, and provides reduced order models with the least response errors as compared with other methods. In addition to that, an advantage that the new approach is applicable to SISO and MIMO discrete type systems.

## 5. Conclusion

In this paper, a robust computational intelligence approach is proposed using GA for MOR of discrete MIMO type systems with the advantage of dominant dynamic preservation. The reduction process is performed based on transforming the system state matrix (in a state space model) while decoupling the multi-time-scale dynamics. The dominant dynamic preservation is performed by retaining the dominant poles of the original system as a subset in the reduced order model utilizing the transformed system state matrix. Once the reduced order model state matrix is designed, the GA MOR new technique will search for the *B*, *C*, and *D* matrices of the reduced order model. Different examples are presented with a comparison of recently published work, such as differential evolution, artificial bee colony, and the Coprime Factor MOR methods. Comparison results show the robustness of the proposed method, as it outperforms such techniques, which points out the potential of the new approach.

## Figures and Tables

**Table 1:** Model order reduction method comparison.

System	Dominant dynamics
Original 8th order	0.8797 ± 0.2442*i*, −0.5875 ± 0.0959*i*
	0.0773 ± 0.1078*i*, −0.0542 ± 0.6558*i*
Proposed 2nd order	0.8797 ± 0.2442*i*
Artificial bee colony [24] 2nd order	0.8778 ± 0.2432*i*
Differential evolution [28] 2nd order	0.8768 ± 0.2484*i*
Genetic algorithm [29] 2nd order	0.8854 ± 0.2398*i*
